# The Book World of Medicine and Science

**Published:** 1900-04-07

**Authors:** 


### THE BOOK WORLD OF MEDICINE AND SCIENCE.

The Medical Annual and Practitioner's Index: A Work of Reference for Medical Practitioners. 1900. Eighteenth year. (Bristol: John Wright and Co. Price 7s. 6d. net.)

Again we have to welcome the appearance of the Medical Annual, a book which the practitioner will find all the more useful in proportion to the regularity with which it is taken in and the extent to which it is made a consulting-room or carriage companion. For the latter purpose it is admirably suited, for while it contains many short articles which are suitable for reference or for scrappy reading, there are plenty of longer ones, the perusal of which may comfortably fill in the break between one patient and the next. Two of the many articles it contains to which we would specially direct attention are those by Major Donald Ross on "Malarial Fever," and by Colonel Keith Hatch on "Mycetoma," the latter of which is admirably illustrated by reproductions of his original coloured drawings. A series of articles on diseases of the stomach must also be referred to, including a full and interesting one by Mr. Walter G. Spencer on the "Surgery of the Stomach," in which a good description is given of the various surgical procedures which are now employed in the treatment of diseases of that organ. Sinusitis is another disease, or group of diseases, which has lately come pretty prominently before the profession, and a very good article on the subject by Dr. Watson Williams will be found useful in explaining the symptomatology and treatment of the various forms of inflammation of the different sinuses connected with the nose. Altogether the book fully maintains its high reputation.

A Manual of Pathology. By Joseph Coats, M.D., late Professor of Pathology in the University of Glasgow. Fourth Edition. Revised throughout by Lewis R. Sutherland, M.B., Professor of Pathology in the University of St. Andrews. With 490 illustrations. (London: Longmans, Green, and Co. 1900. Price 31s. 6d.)

A manual of pathology, more perhaps than one of any other science, requires revision every few years, so rapidly are the increments of knowledge which have to be incorporated with what is already known. But when the main lines of a subject have been laid down, as has been the case in this instance, by one who had devoted his life not only to the advancement, but to the teaching of pathology, new facts fall into their proper places without difficulty, and thus although throughout this book we find traces of the hand of the revisor, in the correction of whatever has been disproved and the addition of whatever has come to light since the publication of the previous edition, the scope and the lines of the book are as of old. The mark of the original author is strong upon it, and although a posthumous edition the work remains a worthy monument to his life and labour.

So thorough and complete is the survey given in this manual of pathology of the morbid life-processes which may affect the body, and of the morbid anatomy, microscopical and macroscopical, which may be demonstrated after death in those affected by disease, that one may almost be tempted by its perusal to question whether the study of pathology ought not to take a somewhat earlier place in the curriculum than it usually does, for although it is true that the more ward work a man gets the better for him, we can hardly doubt that the more carefully a man fresh from his physiology studies pathology in the post-mortem room and in the laboratory, the more fruitful will be his clinical studies in the wards, and the more scientific a practitioner will he ultimately make.

The first part of the book begins with a chapter on the nature, causation, and terminations of disease, in which it is shown that while in its production some external cause is almost always present, the powers of resistance in the tissues are an important element, and that these tissue conditions, if one may so call them, may be either the result of inheritance or may themselves in turn have been set up by the operation of external causes.

Then we have chapters on teratology, on morbid changes in the blood, and in those resulting from disordered circulation, on necrotic, atrophic, and degenerative changes of tissue, and lastly, on the great subjects of inflammation, new formation, tumours, intoxications, and infections. All this is admirably done, and, indeed, forms one of the best of introductions to the systematic study of medicine.

The second part of the book is devoted to diseases of the special organs and systems. As a matter of course this is principally descriptive, but the clinical bearings of the pathological changes are not neglected, so that the reader by no means finds himself studying a mere catalogue of morbid specimens. Everywhere the morbid processes are compared with the changes they have produced, and *per contra*, in describing structural changes mention is also made of the disturbances of function to which they lead. Thus we have a very readable as well as an instructive book. Take it all round, this work is one which, while well up to date, is eminently safe and trustworthy. It is one which can be strongly recommended, not only as a handbook to post-mortem study, but as a useful companion to those who are working at general medicine, whether in the study or in the wards. As a preface to this edition, Sir William T. Gairdner has written an appreciative notice of the life and work of Dr. Coats.

Heart Disease; with Special Reference to Prognosis and Treatment. By Sir William H. Broadbent, Bart., M.D., F.R.S., F.R.C.P., &c., and John F. H. Broadbent, M.A., M.D., M.R.C.P. Third edition. (London: Baillière, Tindall, and Cox. 1900. Price 12s. 6d. net.)

This new edition of Sir William Broadbent's well-known work has been improved and rendered much more complete by the addition of chapters on certain acute affections of the heart, such as endocarditis, pericarditis, and myocarditis, all of which are the work of Dr. John Broadbent, as well as one on aneurysm of the arch of the aorta, which has been compiled by him from notes and papers already published by Sir William Broadbent. We have already favourably reviewed an earlier edition of this book, which was originally based upon certain lectures delivered before the Royal College of Physicians and the Harveian Society. With the addition now made it fairly covers the whole subject, and must take high rank as an authoritative exposition of the main facts concerning the diagnosis, prognosis, and treatment of the various forms of heart disease. The subject is dealt with throughout from the clinical standpoint, and a great deal of practical and useful information is given on the many interesting questions of treatment and prognosis which constantly arise in the daily round of professional work. It is essentially a practitioner's book.

